# Incipient ecological speciation between successional varieties of a dominant tree involves intrinsic postzygotic isolating barriers

**DOI:** 10.1002/ece3.2867

**Published:** 2017-03-14

**Authors:** Elizabeth A. Stacy, Bhama Paritosh, Melissa A. Johnson, Donald K. Price

**Affiliations:** ^1^ Department of BiologyUniversity of Hawai'i HiloHiloHIUSA; ^2^ Tropical Conservation Biology and Environmental Science Graduate ProgramUniversity of Hawai'i HiloHiloHIUSA; ^3^ Present address: School of Life SciencesUniversity of Nevada, Las Vegas4505 S Maryland PkwyLas VegasNV89154USA; ^4^ Present address: Department of BotanyClaremont Graduate University, Rancho Santa Ana Botanic Garden1500 N. College Ave.ClaremontCA91711USA

**Keywords:** fertility, Hawai'i, hybrid fitness, intraspecific, *Metrosideros*, postzygotic reproductive isolation, variety, woody species

## Abstract

Whereas disruptive selection imposed by heterogeneous environments can lead to the evolution of extrinsic isolating barriers between diverging populations, the evolution of intrinsic postzygotic barriers through divergent selection is less certain. Long‐lived species such as trees may be especially slow to evolve intrinsic isolating barriers. We examined postpollination reproductive isolating barriers below the species boundary, in an ephemeral hybrid zone between two successional varieties of the landscape‐dominant Hawaiian tree, *Metrosideros polymorpha*, on volcanically active Hawai'i Island. These archipelago‐wide sympatric varieties show the weakest neutral genetic divergence of any taxon pair on Hawai'i Island but significant morphological and ecological differentiation consistent with adaptation to new and old lava flows. Cross‐fertility between varieties was high and included heterosis of F_1_ hybrids at the seed germination stage, consistent with a substantial genetic load apparent within varieties through low self‐fertility and a lack of self‐pollen discrimination. However, a partial, but significant, barrier was observed in the form of reduced female and male fertility of hybrids, especially backcross hybrids, consistent with the accumulation of genetic incompatibilities between varieties. These results suggest that partial intrinsic postzygotic barriers can arise through disruptive selection acting on large, hybridizing populations of a long‐lived species.

## 1 Introduction

Disruptive selection imposed by heterogeneous environments can lead to phenotypic divergence of populations and the evolution of reproductive isolating barriers within species (i.e., ecological speciation; Rundle & Nosil, 2005; Nosil, 2012). Ecological speciation is expected to involve extrinsic postzygotic barriers (i.e., selection against hybrids) wherever gene flow occurs, unless suitable, typically intermediate, habitat is available for hybrids (Andrew & Rieseberg, 2013). If the differences between diverging populations include heritable traits important for ecological adaptation, then selection against hybrids in parental environments should be proportional to the strength of divergent selection, such that when and where selection wanes, extrinsic postzygotic isolation should also wane (Grant & Grant, 1996; Rundle & Whitlock, 2001; Turelli, Barton, & Coyne, 2001).

In contrast, the role of intrinsic postzygotic barriers in ecological speciation is less clear. Intrinsic postzygotic barriers result when divergent selection or drift in populations in contrasting environments favors different mutations that are incompatible in hybrids (i.e., Agrawal, Feder, & Nosil, 2011; Dettman, Sirjusingh, Kohn, & Anderson, 2007; Funk, Nosil, & Etges, 2006; Gavrilets, 2004). Intrinsic postzygotic barriers can arise through persistent disruptive selection in the face of gene flow under certain conditions (Agrawal et al., 2011; Turelli et al., 2001) and have been documented for a range of ecologically diverged species pairs (e.g., Ålund, Immler, Rice, & Qvarnström, 2013; Bomblies & Weigel, 2007; Fuller, 2008; Kruuk, Gilchrist, & Barton, 1999; Rieseberg, Whitton, & Gardner, 1999; Rogers & Bernatchez, 2006). As most studies of hybrid incompatibilities involve highly diverged species pairs for which isolating barriers are already numerous and strong, however, it is not clear whether the incompatibilities typically arise early in divergence or after speciation is complete (Martin & Willis, 2007; Schemske, 2000). The snowballing nature of genetic incompatibilities expected during population divergence suggests that partial intrinsic postzygotic isolation may evolve quickly (Orr & Turelli, 2001; Sasa, Chippindale, & Johnson, 1998), possibly even before significant differentiation of phenotypic characters (Orr, 1995). Additionally, a large number of studies of outbreeding depression within plant and animal species (Côte, Roussel, Le Cam, & Evanno, 2014; Gimond et al., 2013; Price & Waser, 1979; Templeton, 1986), often across environmental gradients or ecotones, suggest that intrinsic postzygotic isolation may be an important component of early‐stage ecological speciation (reviewed by Scopece, Lexer, Widmer, & Cozzolino, 2010; Aspi, 2000; Pinheiro et al., 2013).

Trees appear to be able to adapt to novel environmental conditions quickly and show significant adaptive divergence across environmental gradients (Savolainen, Pyhäjärvi, & Knürr, 2007); however, the accumulation of reproductive isolating barriers between diverging tree populations is slow (Petit & Hampe, 2006). Trees typically occur in large populations and have a high capacity for long‐distance gene flow (Hamrick, Godt, & Sherman‐Broyles, 1992; Petit & Hampe, 2006; Savolainen et al., 2007). As such, and in contrast with short‐statured and short‐lived plants for which ecological isolation of populations is often associated with geographic isolation, trees in contrasting habitats are far less likely to be allopatric as defined by the absence of gene flow between populations (i.e., migration ≈ 0; Hamrick et al., 1992). Combined, these traits should limit population divergence via genetic drift in trees. Further, given the high genetic load typical of trees, the vast majority of tree species is nearly obligately outcrossed (Petit & Hampe, 2006). Lastly, because of their long generation times, trees can experience dramatic changes in the strength and direction of selection within a lifespan (Petit & Hampe, 2006). These characteristics should slow the evolution of reproductive isolating barriers, and they likely contribute to lower speciation rates in trees relative to other plants.

*Metrosideros polymorpha* vars. *incana* and *glaberrima* (hereafter *incana* and *glaberrima*) are the most abundant and widespread of the many taxa within the landscape‐dominant tree genus *Metrosideros* in Hawai'i, whose progenitor likely colonized the oldest (5 myo) main island of Kaua'i roughly 4 mya (Percy et al., 2008). Whereas many *Metrosideros* taxa are single‐island endemics, *incana* and *glaberrima* are the only forms found on all of the main islands, with populations on young Hawai'i Island derived from those on older islands (Percy et al., 2008; Stacy, Johansen, Sakishima, Price, & Pillon, 2014). On young (<700,000 years old; Ziegler 2002), volcanically active Hawai'i Island, *incana* and *glaberrima* are successional varieties (or ecotypes, Turesson, 1922) that dominate new and old lava flows, respectively, at low to middle elevations (Drake & Mueller‐Dombois, 1993; Stemmermann, 1983). The contrasting adult distributions of these varieties on active volcanoes coincide with significant between‐variety differences in plant and leaf morphology, leaf nutrient content and water retention, seed germination responses to light and heat, and seedling‐stage responses to light and soil nitrogen, all of which are consistent with differential adaptation to the harsh and relatively benign abiotic conditions of new and old substrates, respectively (Dawson & Stemmermann, 1990; Drake, 1993; Kitayama, Pattison, Cordell, Webb, & Mueller‐Dombois, 1997; Morrison & Stacy, 2014; Stacy, Johansen, Sakishima, & Price, 2016; Stemmermann, 1983; Vitousek, Turner, & Kitayama, 1995). Common garden studies and parent–offspring analysis have revealed a heritable basis for several leaf morphological (Stacy et al., 2016; Stemmermann, 1983) and functional (Kitayama et al., 1997) traits. Both varieties produce typically red, showy shaving‐brush inflorescences visited by birds and insects (Carpenter, 1976; Corn, 1979; Koch & Sahli, 2013) and dry capsules with tiny wind‐borne seeds (Drake, 1993). *Incana* and *glaberrima* show the weakest pairwise divergence (F_ST_ = 0.05) of any pair of *Metrosideros* taxa on Hawai'i Island (DeBoer & Stacy, 2013; Stacy et al., 2014) and appear to be maintained at a weak stage of divergence through recurring introgression on intermediate‐aged lava flows.

Due to a lack of apparent premating barriers between these varieties, ephemeral intraspecific hybrid zones form readily on intermediate‐aged lava flows at low‐to‐middle elevations on Hawai'i Island. Complete successional replacement of *incana* by *glaberrima* on aging lava flows takes from 1,400 to 3,000 years depending on substrate type (Drake & Mueller‐Dombois, 1993; Kitayama, Mueller‐Dombois, & Vitousek, 1995), allowing an estimated 30–50 generations of introgression between the varieties and their fertile hybrids with each new flow (Stacy et al., 2016). The high abundance of hybrid trees on intermediate‐aged lava flows suggests high hybrid growth and survivorship through to the adult stage, consistent with the geographically bounded hybrid superiority model of hybrid zones (Stacy et al., 2016). The abundance of hybrids on intermediate‐aged lava flows also indicates that these forms remain at an early stage of the speciation continuum (Nosil, 2012) even on young Hawai'i Island.

In the *incana‐glaberrima* hybrid zone on the 1855 Mauna Loa flow on east Hawai`i Island, the variation in adult leaf pubescence at the site and the consistency between adult phenotypes and those of offspring of controlled crosses among hybrid zone trees suggest the presence of two hybrid classes, F_1_s and *incana* backcrosses (Stacy et al., 2016). Such a concentration of early generation hybrids would be expected on a lava flow that has supported only a few generations. Here, we report the results of hand crosses among adults of *incana*,* glaberrima,* and their hybrids on the 1855 flow to uncover what intrinsic postzygotic barriers, if any, maintain the incipient boundary between these ecologically diverged varieties. Lastly, postpollination prezygotic isolation between the varieties was also examined as well as self‐fertility and pollen limitation of all tree classes.

## 2 Methods

### 2.1 Study site

This study was conducted at ~880 m above sea level (near mile markers 11 and 12 on Saddle Road) on the 1855 Mauna Loa lava flow on east Hawai'i Island, where *M. polymorpha* forms a monospecific stand (described in a companion paper, Stacy et al. (2016)). The 1855 Mauna Loa lava flow is surrounded by 3,000‐ to 5,000‐year‐old substrate supporting mature rainforest dominated by late‐successional *glaberrima*. Because of the lava flow's intermediate age and the high interfertility between varieties, the site hosts a mixed population of early successional *incana*, late‐successional *glaberrima*, and their hybrids. Analysis of adult morphology in the hybrid zone and parent–offspring analysis of 2‐year‐old seedlings produced through crosses among hybrid zone trees strongly support the presence of F_1_ and backcross‐*incana* trees at the site (Stacy et al., 2016). Adults were designated to type by leaf pubescence [permanent leaf pubescent = *incana*; glabrous (pubescence absent) = *glaberrima*; nonpermanent (caducous) leaf pubescence easily removable by rubbing = F_1_ hybrids; and nonpermanent leaf pubescence removable by vigorous rubbing = backcross‐*incana*]. The classification of trees to type by this method was fully consistent among project personnel. We use these designations throughout this article, acknowledging the possibility of misclassification of a presumably small fraction of the hybrid trees. The young substrate and open canopy on the 1855 flow led to the short stature of many adult trees and allowed access to flowers for hand pollinations that at other sites are out of reach. Seventy‐two flowering adults representing all four tree classes were selected haphazardly from across a ~1,200‐m by 200‐m area for controlled hand pollinations.

### 2.2 Intrinsic postzygotic reproductive isolation

From May through August in 2006 and 2007, 72 trees were used for reciprocal hand crosses. The crossing design, hand pollinations, and processing of resulting fruits and seeds are described in Stacy et al. (2016). Briefly, crosses were performed within and between all four tree types to allow examination of cross‐fertility between the two successional varieties, and fertility of all forms. Each tree was crossed reciprocally (i.e., as both maternal tree and pollen donor) with each of two other trees having contrasting phenotypes. Selection of adults was haphazard from among flowering trees within each type and resulted in experimental crosses involving 30 *glaberrima*, 13 *incana*, and 29 hybrid adults (15 and 14 F_1_ and backcross‐*incana* hybrids, respectively). Fewer *incana* adults were available for study due to the greater height of these early successional trees (Stacy et al., 2016), and two experimental trees of *incana* were lost to road widening part way through the study. Each cross involved ≥20 emasculated flowers from >1 inflorescence on a maternal tree protected by a mesh pollinator‐exclusion bag. Parent–offspring analysis of vegetative traits of the resulting 2‐year‐old seedlings is presented elsewhere (Stacy et al., 2016). Here, we report data on reproductive isolating barriers observed using those same crosses.

#### 2.2.1 Fruit set

For each experimental inflorescence, the final count of nascent fruits remaining upon removal of the pollinator‐exclusion bag (14 days postpollination) was used as initial fruit set for calculation of fruit set rate. Ripening fruits were counted on each experimental infructescence every month and collected when mature (sutures on the dehiscing capsules become discolored). The numbers of initial fruits and mature fruits were each summed across inflorescences within each cross on each maternal tree and used to calculate the final fruit set rate (final fruit number/initial fruit number). For each hand‐pollinated inflorescence that produced mature fruits, the duration of the fruit maturation period was calculated as the number of days between pollination and collection of the first mature fruit. Fruit set data provided information on female fertility of parental variety and hybrid trees as well as cross‐fertility among tree types.

#### 2.2.2 Seed germination

Seeds from mature experimental fruits (≤10 per cross) were sown (one fruit per two, 5 × 10 cm wells) atop well‐draining media (one part super‐coarse perlite, two parts sifted cinder, and three parts Sun Gro Horticulture Sunshine Mix #1) covered with a thin layer of black sand to prevent loss of the tiny seeds into cracks from which they may not emerge (Drake, 1993). Seedling trays were placed in a mist house set to water every 10 min for five seconds during daylight hours (~50% sunlight) and remained there for 8 weeks. The number of germinants per fruit was recorded every 2 weeks for 6 weeks. Recounts of a random subset of fruits at 8 weeks indicated that the vast majority of seeds had germinated by 6 weeks. The number of germinants per fruit was averaged across fruits from each cross within each maternal tree, and means based on fewer than three fruits (*n* = 18 crosses) were excluded from the analysis of seed germination rate (however, the excluded multiseeded fruits were retained to produce seedlings for growth measurements). Seed germination data provided information on female fertility of parental variety and hybrid trees as well as cross‐fertility among tree types.

#### 2.2.3 Seedling growth in the greenhouse

After 8 weeks in the misthouse, seedling trays were moved to a plastic coldframe structure with screened sides, ~90% sunlight (maximum), and overhead watering 3× per day for 3–5 min each (adjusted as needed). Environmental conditions (e.g., cloud cover, day length, temperature, humidity) were otherwise identical to ambient conditions at ~100 m above sea level on east Hawai'i Island. Seedling trays were rotated within the greenhouse monthly, and at about 3 months postgermination, seedlings were thinned to leave a maximum of six well‐spaced seedlings per 5 × 10 cm well. Eight months after germination, seedlings were measured for height (shoot length, in cm) to apical meristem and number of leaf nodes. A subset of seedlings (mean = 13) from each family (i.e., maternal tree × pollen donor combination) was then transplanted into individual 10‐cm pots. Two seedlings were randomly selected for transplanting from the six in each communal well, as needed, using a six‐sided dice. Transplanted seedlings were measured for height again at 2 years of age, and relative growth rate (RGR) over 16 months was calculated for each seedling as: RGR = $\frac{\ln\left(H_{24}\right)-\ln\left(H_{8}\right)}{16}$, where *H*_24_ and *H*_8_ are height at 24 and 8 months, respectively. Light fertilizer and pesticides were applied evenly as needed over the 2 years. Seedling growth data provided information on fitness variation among parental‐variety and hybrid seedlings under benign conditions.

#### 2.2.4 Hybrid male fertility

Given the utility of pollen stainability as a measure of hybrid fertility (e.g., Fishman & Willis, 2001; Mayer, 1991), we contrasted pollen stainability among the four tree types in the hybrid zone using cotton blue‐lactophenol, which stains starch in intact gametophytes (Kearns & Inouye, 1993). The percentage of normal (i.e., fully formed, darkly stained) versus aborted or abnormal pollen grains (i.e., empty or only partly filled) was estimated from a sample of ≥300 pollen grains from each of approximately 20 adults of each of the four tree types. Selection of additional trees for examination of pollen stainability was haphazard among flowering trees at the site and involved a pole pruner (for *incana*).

### 2.3 Postpollination prezygotic isolation

#### 2.3.1 Pollen tube growth

Supplemental hand pollinations were performed to allow examination of possible reproductive isolation by reduced pollen tube growth in between‐variety crosses. The absence of a significant pollen donor type‐by‐maternal tree type interaction effect on either fruit set or seed germination (see Results) indicated that pollen tube growth was an unlikely barrier between varieties. Consequently, examination of pollen tube growth in outcrossed styles was restricted to the reciprocal crosses of three *incana* × *glaberrima* pairs. Within‐variety crosses were performed for comparison (*n* = 3 per each of the two varieties) using the same six maternal trees. Ten days after hand pollination, styles from these 12 crosses (five styles per cross) were collected and placed in 70% ethanol. Styles were stained with decolorized aniline blue, squashed (Kearns & Inouye, 1993), and examined at 100× under a fluorescent microscope. Slides were examined “blind” to cross‐type, labeled with only alphanumeric codes. As the pollen tube bundles examined were uniformly robust, variation in bundle density was not recorded. Rather, the length (mm) of the pollen tube bundle was recorded for each style examined.

### 2.4 Self‐fertility and pollen limitation

Any additional inflorescences on the experimental trees were used for tests of self‐fertility and pollen limitation. Emasculated and bagged inflorescences were pollinated with self‐pollen (*n* = 66 trees), and additional inflorescences were tagged and unmanipulated for estimation of natural rates of fruit set and seed germination (open‐pollination; *n* = 67 trees); not all pollination treatments were possible on all 72 trees due to limited inflorescences. A minimum of 20 flowers/maternal tree from multiple inflorescences was used for each of the selfed and open‐pollination treatments. For selfed treatments, fruit set, seed germination, and seedling growth in the greenhouse were recorded as above for outcrossed treatments. Open‐pollinated seedlings were discarded after fruit set and seed germination data were recorded. Lastly, to examine discrimination of self‐pollen, we contrasted pollen tube growth between self‐pollinated and out‐crossed flowers (following the methods above) on additional paired self and outcross pollinations. Simultaneously, self‐pollen tube growth was compared between self‐fertile and self‐infertile trees (as determined by fruit set of selfed flowers), as follows. Thirteen *glaberrima* pollen donors were used in single‐donor outcrosses on 18 of the maternal trees (nine *glaberrima*, two *incana*, seven hybrid); nine of these maternal trees produced no fruits from selfed pollinations, and nine of these trees had nonzero fruit set from selfed flowers. At least five styles from each of the 36 pollinations were harvested 10 days after pollination for fluorescent microscopy. The pollen tube bundles examined were again uniformly robust, so only bundle length (mm) was recorded for each style examined. For each of 65 trees for which both data types were available, a pollen‐limitation index was calculated as: *L* = 1 − (*P*_O_/*P*_S_), where *P*_O_ and *P*_S_ are % fruit set of open‐ and cross‐pollinated flowers, respectively (Larson & Barrett, 2000).

### 2.5 Statistical analyses

*(Reproductive isolation)*: Dependent variables were tested for normality and equal variances and normalized as necessary using Johnson's transformation (see Stacy et al., 2016; Minitab Inc. Pennsylvania, PA, USA; Johnson, 1949; Chou, Polansky, & Mason, 1998). To examine the significance of postzygotic isolating barriers among tree types, two‐way ANOVAs were conducted to determine the influence of maternal tree type, pollen donor type (treated as fixed factors), and their interaction on each of mean fruit set rate, mean fruit maturation time, mean number of germinants per fruit, mean number of leaf nodes at 8 months, mean seedling height at 8 and 24 months, and mean RGR over 16 months. Where only maternal tree‐type effects were found, follow‐on one‐way ANOVAs were performed to contrast response variables across maternal tree types, but with data averaged across all outcrossed inflorescences (combining the two pollen donors) per maternal tree. Seed germination rates were further compared between the within‐variety and between‐variety crosses using a Mann–Whitney *U*‐test, and the timing of seed germination was compared across tree classes using ANOVA of the proportion of total germinants that had germinated by week 2. The mean proportion of normal pollen (of 300 grains examined per tree) was compared among tree types using a one‐way ANOVA. Tukey's multiple comparison tests (*p* < .05 family error rate) were carried out for all significant response variables in one‐way ANOVAs. Pollen tube bundle lengths from the 12 paired within‐ and between‐variety crosses were compared using a one‐way *t*‐test, such that H_0_: mean between‐variety pollen tube length/mean within‐variety pollen tube length = 1. *(Self‐fertility and Pollen limitation):* Using the subset of 62 trees on which all three pollination treatments were performed, the above outcrossed treatments were compared with selfed and open‐pollinated treatments at both the fruit set and seed germination stages in a randomized block design using two‐way ANOVA with maternal tree (random factor) and pollination type (block) as predictor variables followed by Tukey's multiple comparison tests. Pollen tube bundle lengths from selfed and outcrossed styles were compared using a one‐way *t*‐test, such that *H*_0_: mean selfed pollen tube length/mean outcrossed pollen tube length = 1. Self‐ and open‐pollinated treatments were compared among tree classes at both the fruit set and seed germination stages using one‐way ANOVAs. For self‐pollination‐derived seedlings, measures of size at 8 months (height and number of leaf nodes) were compared among tree classes using ANOVA with Tukey's multiple comparisons.

## 3 Results

### 3.1 Intrinsic postzygotic reproductive isolation

#### 3.1.1 Fruit set

**Figure 1:**
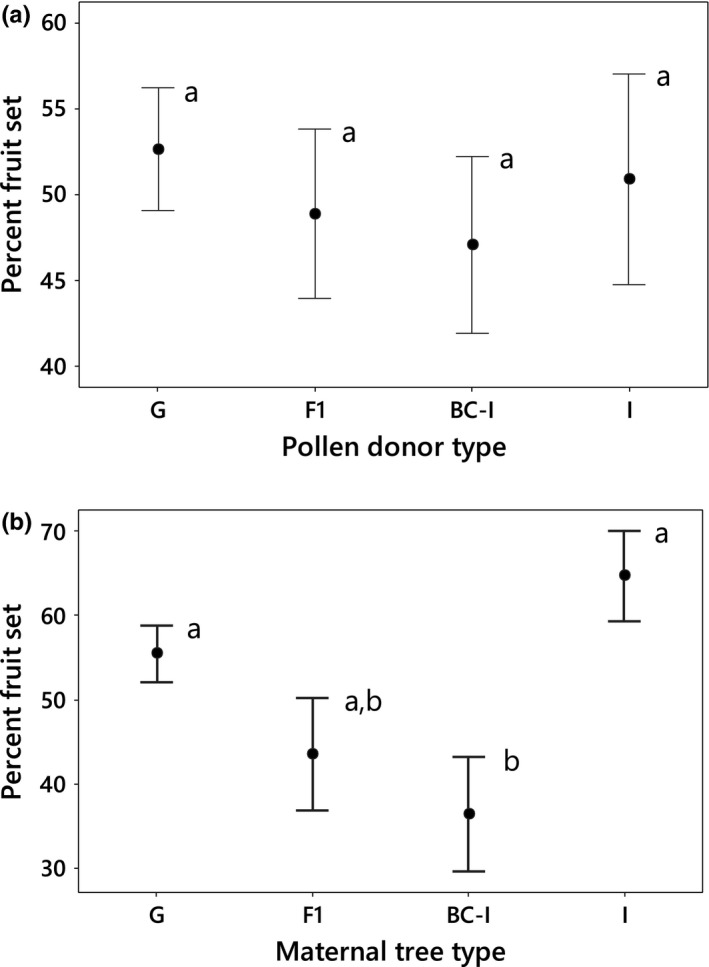
Mean (±1 SE) percent fruit set resulting from experimental crosses within and among four tree types at the study site. Data are shown for 30 trees of *glaberrima* (G), 15 F_1_ trees (F1), 14 backcross‐*incana* trees (BC‐I), and 12 trees of *incana* (I) grouped by (a) pollen donor type and (b) maternal tree type. Groups with shared superscripts are not significantly different at α = .05

The percentage fruit set was recorded for 138 outcrosses involving 72 trees (mean = 25.6 flowers/cross; 3,578 flowers total). There was no significant pollen donor‐type effect on fruit set (Figure 1a), nor significant interaction effect of maternal tree and pollen donor type (two‐way ANOVA of % fruit set bounded Johnson transformed: *p* > .05). However, fruit set was significantly lower for maternal trees of backcross‐*incana* (36.34 ± 6.79 (SE) %) relative to those of *incana* (64.71% ± 5.37%) and *glaberrima* (55.48% ± 3.36%; one‐way ANOVA: F_3,67_ = 4.77, *p* = .005, R^2^ = 13.9%); fruit set of F_1_ hybrids was intermediate (43.52% ± 6.68%; Figure 1b).

The duration of the fruit maturation period varied across inflorescences from 165 to 291 days (mean ± SE: 218.3 ± 3 days). There was no significant pollen donor type effect nor significant interaction effect of maternal tree and pollen donor type on fruit maturation time (two‐way ANOVA: *p* > .05). However, mean fruit maturation time was significantly shorter for maternal trees of *incana* (199.2 ± 5.0 (SE) days) than for maternal trees of *glaberrima* (226.2 ± 5.0) or F_1_ hybrids (224.2 ± 6.26; F_3,64_ = 4.55, *p* = .006, R^2^ = 13.7%); the duration of the fruit maturation period for backcross‐*incana* hybrids (209.7 ± 4.35; Fig. S1) was intermediate and trending closer to *incana*.

#### 3.1.2 Seed germination

Seed germination was recorded for 957 fruits from 120 experimental outcrosses. There was no significant maternal or paternal tree class effect or interaction effect (two‐way ANOVA: *p* > .05) on the mean number of germinants per fruit. Across maternal tree types, the mean number of germinants per fruit ranged nonsignificantly from 74.13 ± 8.96 (SE) for F_1_ hybrids to 111.1 ± 12.9 for *incana*.

**Figure 2:**
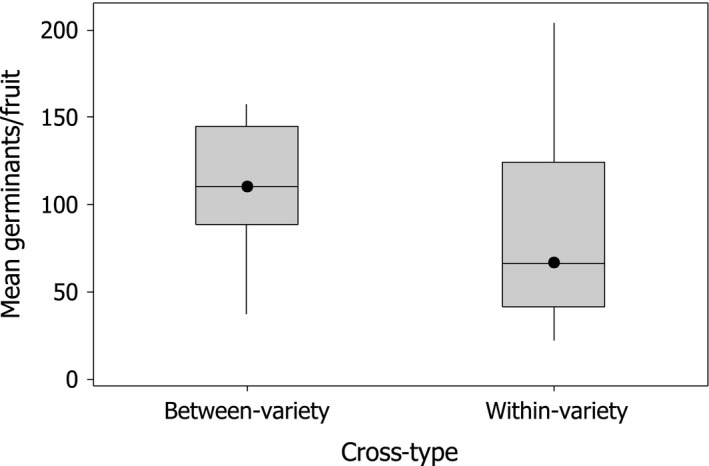
Median, quartiles, and range of the number of germinants per fruit for 39 independent crosses between and within *glaberrima* and *incana*;* n* = 12 and 27 between‐ and within‐variety crosses, respectively. Mann–Whitney *U*‐test: *U* = 5.0, *p* = .025

Comparison of seed germination rates of within‐ versus between‐variety crosses (i.e., with hybrid parents removed) was also carried out. Between‐variety crosses revealed a 40% and 45.7% increase in the number of germinants per fruit for maternal trees of *glaberrima* and *incana*, respectively, compared to within‐variety crosses (median between variety = 110.4, median within variety = 66.3; Mann–Whitney *U*‐test of seed germination at 6 weeks: *U* = 5.0, *p* = .025, *n* = 12 and 27 independent between‐ and within‐variety crosses, respectively; seven nonindependent within‐variety crosses were removed from the analysis; Figure 2). Seed germination rates were similar between varieties for both the within‐variety crosses and between‐variety crosses and were pooled for the Mann–Whitney *U*‐test.

Seed germination began 5–7 days after sowing for all but one tree (backcross‐*incana* tree # 10) whose seeds initiated germination within 3 days. Regardless of pollen donor, seeds from maternal trees of *incana* germinated more quickly than those of *glaberrima*, and those of hybrids were intermediate in germination time; the proportion of total seedlings that had germinated at 2 weeks was 77.6 ± 8.0 (SE) % for maternal trees of *incana*, 48.9% ± 4.8% for *glaberrima*, 53.2% ± 9.7% for F_1_ hybrids, and 54.6 ± 6.0 for backcross‐*incana* hybrids; F_3,65_ = 2.8, *p* = .047, R^2^ = 7.36 (Fig. S2).

#### 3.1.3 Seedling growth in a greenhouse: (8 months)

At 8 months, 3,191 seedlings from 126 cross pollinations were measured. The mean height of outcrossed seedlings was not influenced by paternal tree type, nor by any maternal tree‐by‐pollen donor type interaction (two‐way ANOVA: *p* > .05). However, seedlings from *incana* maternal trees (4.26 ± .39 (SE) cm) were shorter than those from *glaberrima* maternal trees (5.32 ± .24 cm), regardless of pollen donor type (F_3,65_ = 2.51, *p* = .067, R^2^ = 6.24%; F_2,66_ = 3.82, *p* = .027, R^2^ = 7.66% with hybrids pooled); seedlings of the two hybrid maternal tree classes were very similar and intermediate in height on average (4.66 ± .20 cm) but overlapped with those of pure‐variety maternal trees. The mean number of leaf nodes (10.9 ± .21 (SE)) did not vary among any of the seedling groups compared.

#### 3.1.4 Seedling growth in a greenhouse: (2 years)

**Figure 3:**
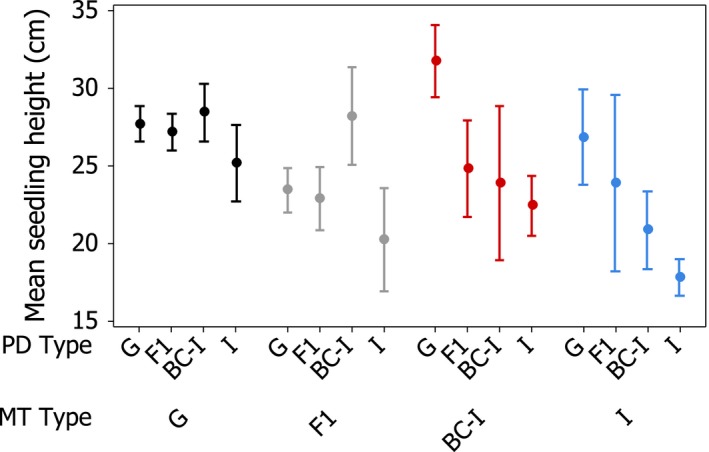
Mean (±1 SE) height at 2 years of outcrossed seedlings produced through crosses among the four tree types; PD Type = pollen donor type, MT Type = maternal tree type (color coded), and tree codes are as in Figure 1. Both maternal tree type (*p* < .026) and pollen donor type (*p* < .011) affected seedling height

Two‐year survivorship of the 1,638 potted‐up seedlings was high (97%) and even across cross types. In contrast to 8‐month measures, seedling height at 2 years was significantly affected by both maternal and paternal tree types (Figure 3). Seedlings of *incana* parents were significantly shorter than those of *glaberrima* parents (two‐way ANOVA: maternal tree type: F_3,109_ = 3.22, *p* = .026; paternal tree type: F_3,109_ = 3.92, *p* = .011; R^2^ = 12.64%; Figure 3), but the interaction term was not significant (*p* = .32). Seedlings of hybrid maternal trees varied in height but were intermediate on average. Mean seedling height was significantly associated with the mid‐parent value for the anticipated proportion of *incana* and *glaberrima* alleles (*p* < .001, R^2^ = 9.7). Compared with seedling heights at 8 months, heights at 2 years suggested a shift in the RGRs of the different genotypes. Differences in seedling height appear to accumulate over time, with the largest initial differences occurring between the parental varieties, *glaberrima* and *incana*. Mean RGR between 8 and 24 months of age showed the same pattern as height at 2 years (two‐way ANOVA: maternal tree type: F_3,107_ = 2.73, *p* = .048; pollen donor type: F_3,107_ = 3.47, *p* = .019, R^2^ = 46.91%). Neither survivorship nor growth of seedlings in the greenhouse indicated any evidence of reduced hybrid fitness due to intrinsic causes; all of the cross types produced some number (1.5%–34.7%; mean = 14.5%) of seedlings that retained stunted, juvenile phenotypes at 2 years of age.

#### 3.1.5 Hybrid male fertility

**Figure 4:**
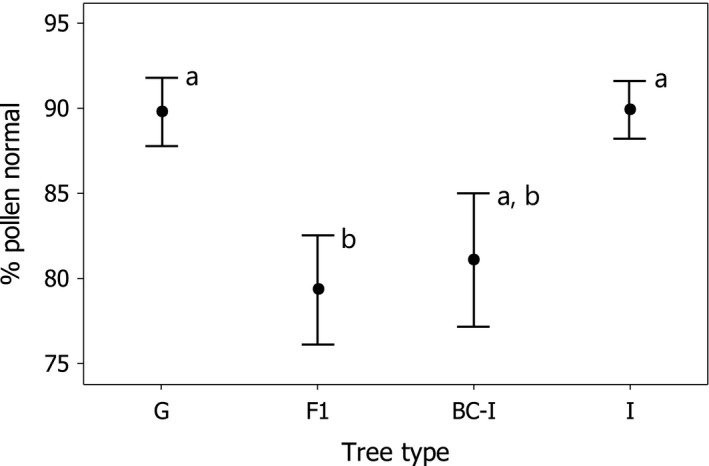
Pollen stainability of two varieties and their hybrids at the study site. Mean (±1 SE) percent normal pollen as detected with cotton blue stain at 40× of 20 trees of *glaberrima* (G), 22 F_1_ hybrids (F1), 19 backcross‐*incana* hybrids (BC‐I), and 21 trees of *incana* (I). Groups with shared superscripts are not significantly different at α = .05

The percentage of pollen that contained fully formed gametophytes was significantly lower for F_1_ hybrids (79.35 ± 3.21) and nearly so for backcross‐*incana* hybrids (81.10% ± 3.94%) than for the parental varieties, which showed similar levels (~90% normal pollen; one‐way ANOVA of % normal pollen: F_3,78_ = 3.99, *p* = .011, R^2^ = 9.99%; Figure 4). Pollen stainability was significantly lower for pooled hybrids relative to both varieties (F_2,79_ = 5.96, *p* = .004, R^2^ = 10.91%).

### 3.2 Postpollination prezygotic reproductive isolation

#### 3.2.1 Pollen tube growth

Pollen tube growth was vigorous in all styles examined, from both within‐variety pollinations and reciprocal crosses between varieties. Of 60 styles examined, the average ratio of pollen tube bundle lengths of paired within‐ and between‐variety styles (.97) was not different from one (1‐sample *t*‐test: T = −.55, *p* = .61, *n* = 6 maternal trees); for this test, within‐variety bundle lengths were averaged for the two maternal trees involved in each pairing. This result, in concert with the hybrid vigor observed at the seed germination stage for the full set of between‐variety crosses, indicates that differential pollen tube growth does not contribute to reproductive isolation between *incana* and *glaberrima* on east Hawai'i Island.

### 3.3 Self‐fertility and pollen limitation

#### 3.3.1 Fruit set

Compared to the mean rate of fruit set of pooled outcrossed flowers on the same trees (50.51 ± 2.85 (SE) %), fruit set of selfed flowers (33.90% ± 3.77%), but not of open‐pollinated flowers (41.39% ± 3.50%), was significantly reduced (F_2,122_ = 7.82, *p* = .001, R^2^ = 27.07%, *n* = 62 trees with all three treatments; Fig. S3A). Neither selfing‐derived fruit set nor open‐pollinated fruit set differed among the varieties and their hybrids. Fruit set from self‐pollinations varied among individuals, however, with five of the 67 self‐pollinated trees failing to produce selfed fruits in both years. The pollen limitation index trended lower (median = 0%) for backcross hybrids relative to the other tree classes (medians: 27.9%–55.2%); however, the difference was not significant (Kruskal–Wallis: *H* = 6, *df* = 3, *p* = .112; Fig. S4).

#### 3.3.2 Seed germination

Seed germination was recorded for 337 fruits from self‐pollinated flowers on 49 trees and 429 fruits from open‐pollinated flowers on 58 trees. The mean number of seeds germinated per fruit at 6 weeks differed significantly among the three pollination treatments, from 94.68 ± 8.46 (SE) germinants/fruit for pooled outcrossed flowers, to 35.70 ± 4.2 germinants/fruit for open‐pollinated flowers, and 13.93 ± 2.64 germinants/fruit for selfed flowers (F_2,84_ = 72.5, *p* < .001, R^2^ = 59.08, *n* = 43 trees; Fig. S3B). Unusually high selfed seed germination was recorded for a single tree (backcross‐*incana* tree #10) that produced an average of 109.7 ± 15.4 germinants per selfed fruit (*n* = 11 fruits). Neither selfed nor open‐pollinated seed germination rates differed among the maternal tree types (*p* > .05).

#### 3.3.3 Early growth of selfed seedlings

**Figure 5:**
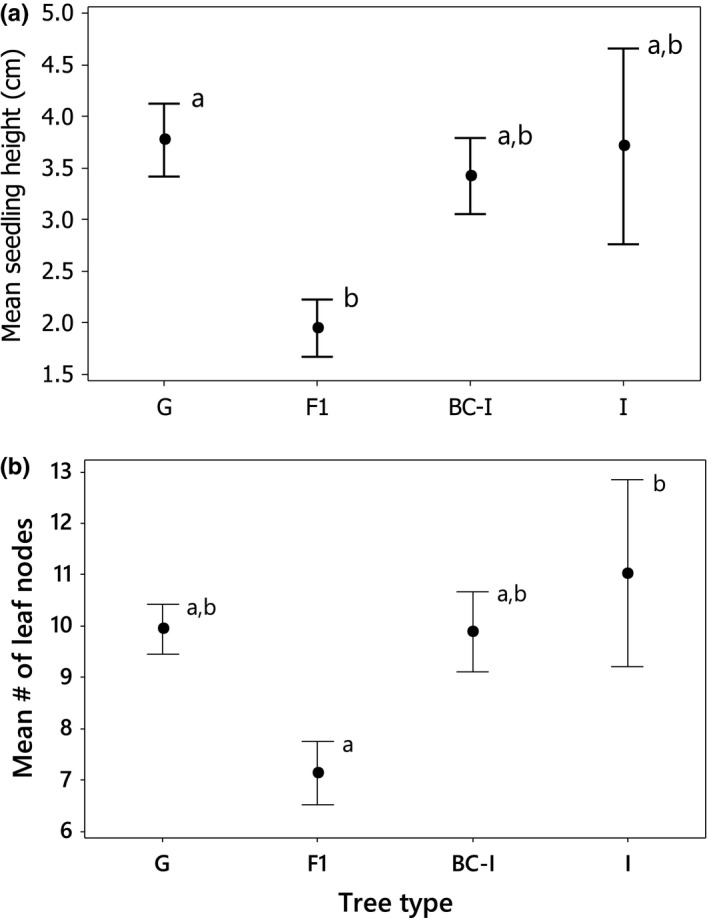
Comparison of 8‐month‐old seedlings produced through self‐pollinations of four types of maternal trees: *glaberrima* (G; *n* = 21), F_1_ hybrids (F1; *n* = 7), backcross‐*incana* hybrids (BC‐I; *n* = 11), and *incana* (I; *n* = 5); (a) mean (±1 SE) seedling height, and (b) mean (±1 SE) number of leaf nodes. Sharing of superscripts indicates no significant difference at α = .05

At 8 months, 603 seedlings from 44 self‐pollinations were measured. Seedlings from self‐pollinations were significantly shorter (3.40 ± .23 [SE] cm) than outcrossed seedlings (4.88 ± .18 cm; paired *t*‐test: T = 5.07, *df* = 76, *p* < .001, *n* = 42 maternal trees with both selfed and outcrossed seedlings). With one exception, there were no differences in either height nor number of leaf nodes among selfed seedlings from different maternal tree types; selfed seedlings from F_1_ maternal trees were significantly stunted compared to other classes of seedlings according to both measures, with a 43%–48% reduction in height and 28%–35% fewer nodes (height unbound Johnson transformed: F_3,40_ = 3.64, *p* = .021, R^2^ = 15.6%; number of nodes: F_3,40_ = 3.03, *p* = .041, R^2^ = 12.4%; Figure 5). This height difference was evident in spite of the apparent impact of inbreeding depression on the heights of selfed seedlings from all maternal tree types; as a percentage of the average height of the within‐variety cross seedlings from the same mother, heights of selfed seedlings averaged 77% ± 8.2% for *glaberrima* (22 maternal trees) and 81.8% for *incana* (1 maternal tree). For F_1_ trees, the average height of selfed seedlings was just 39.3% ± 17% (3 maternal trees) that of outcrossed seedlings.

#### 3.3.4 Pollen tube growth

Of 214 styles examined, the average ratio of pollen tube bundle lengths of paired selfed and outcrossed styles (1.14) was not different from one (1‐sample *t*‐test: T = 0.72, *p* = .484, *n* = 18), suggesting a lack of discrimination of self pollen in any tree examined.

## 4 Discussion

This study examined several measures of adult and cross fertility in an intraspecific hybrid zone comprising two successional varieties of a landscape‐dominant tree. The results reveal similar levels of self‐ and natural fertility among the parental varieties and their hybrids, but significantly reduced hybrid pollen stainability and female fecundity of hybrids when supplemented with outcrossed pollen. Reduced hybrid fertility, which appears to be the sole intrinsic postpollination isolating barrier present between the two successional varieties, likely arose as a consequence of differential adaptation of these varieties to contrasting environments (Hereford, 2009; Rundle & Whitlock, 2001).

### 4.1 Mixed mating system and high genetic load

*Incana* and *glaberrima* appear to have a mixed mating system characterized by predominant outcrossing with the ability to self‐fertilize retained in many trees. Self‐fertility was limited by a substantial genetic load apparent in the stunting of selfed seedlings and the reduced fruit and seed set in self‐pollinated treatments coupled with the robust growth of pollen tubes in selfed treatments. Mixed mating systems with predominant outcrossing are known in the close relative, *Metrosideros excelsa*, of New Zealand (Schmidt‐Adam, Gould, & Murray, 1999) and are common in eucalypts (also in family Myrtaceae), where low self‐fertility is caused frequently by inbreeding depression (see Burrows, 2000; Potts & Savva, 1988). If late‐acting self‐incompatibility (i.e., abscission post fertilization; Seavey & Bawa, 1986), which is known in Myrtaceae (e.g., Finatto et al., 2011), plays a role in the low self‐fertility seen in *M. polymorpha*, it varied significantly among individuals within both varieties. Low self‐fertility due to high genetic load is common in trees, where predominant outcrossing, large populations, and long lifespans facilitate the accumulation of mutations (Petit & Hampe, 2006). The significant hybrid vigor of F_1_ seeds observed in this study is consistent with contrasting loci underlying part of the substantial genetic load observed in both varieties and partial reproductive isolation maintaining these differences.

### 4.2 Ecological divergence and the evolution of incompatibilities

*Incana* and *glaberrima* differ in a number of phenotypic and life history traits that appear consistent with their contrasting successional roles on active volcanoes. Early successional *incana* has greater water retention (Stemmermann, 1983), a shorter fruit maturation period (this study), and more rapidly germinating seeds than *glaberrima* (Drake, 1993; this study), which may reflect adaptation to the more ephemeral moisture availability of new lava flows (Drake, 1993). Further, these varieties differ in leaf nitrogen content (Vitousek et al., 1995) and show significant divergence at the seedling stage along light‐availability and (to a lesser extent) nitrogen‐availability gradients, exhibiting the classic plant life history tradeoff between fast growth in high light (*incana*; 60% greater total dry mass) and high survivorship in shade (*glaberrima*; 76% greater survivorship; Morrison & Stacy, 2014). Also consistent with the different successional statuses of these varieties is the contrasting pattern of resource allocation observed in seedlings with early‐successional *incana* investing more in root growth than shoot growth, and late‐successional *glaberrima* showing the opposite pattern (Morrison & Stacy, 2014), reflected in the current study in the significant negative correlation between height at 2 years and the proportion of *incana* alleles in seedlings. This difference in resource allocation may reflect adaptations to depth of available water in the two habitats (Stemmermann, 1983).

The only postpollination reproductive barrier observed between *incana* and *glaberrima* in this study was a partial, but significant, reduction in hybrid fertility. Low hybrid fertility was manifest in fruit set in controlled crosses (28%–38% reduction in fruit set of pooled hybrids compared to that of maternal trees of the two parental varieties) and pollen stainability (11% reduction). The latter could be viewed as a conservative estimate of male hybrid fertility, given that cotton blue allows determination of fully formed versus abnormal male gametophytes only, and some fully formed gametophytes may be unviable. Fruit set and seed germination rates of open‐pollinated treatments did not differ across tree types, and seedling growth varied by variety only (with hybrids intermediate). Comparison across the four tree types revealed a trend of lower pollen limitation in backcross‐*incana* hybrids due solely to their lower fruit set in the cross‐pollination treatment. Thus, natural female fertility did not vary across tree types—only reproductive capacity reached through supplemental pollinations. Interestingly, the decreased pollen stainability of hybrids did not translate into reduced fruit set or seed germination in crosses with hybrid pollen donors. This may be because any impact of a modest loss in pollen viability of hybrids was swamped out by the high artificial pollen load used in the hand pollinations (i.e., fruit and seed production were likely more limited by the number of ovules that could ripen). Comparable hand pollinations in a related species, *M. excelsa*, of New Zealand revealed an average of >618 pollen grains germinating on hand‐pollinated stigmas, yet only one‐third of the >933 (average) ovules per flower typically set seeds (Schmidt‐Adam et al., 1999). It is possible that hybrid pollen is less competitive than pollen from the parental varieties when delivered to stigmas in mixed‐donor loads (e.g., Campbell, Alarcon, & Wu, 2003), which are likely more representative of natural pollinations by insects and birds (Harder & Barrett, 1996). Even if our working model of hybrid genotypes is not correct (i.e., if some of our F_1_ and backcross‐*incana* hybrids are misclassified), these results reveal that reduced hybrid fertility is a significant isolating barrier between these varieties. Despite the reduced fitness of *incana‐glaberrima* hybrids, the nonzero fertility of backcrossed trees allows introgression between varieties and is consistent with the weak neutral genetic differentiation between these taxa in spite of their significant adaptive differences.

The observed fitnesses of F_1_ and backcross trees suggests a role for Dobzhansky‐Muller incompatibilities (DMIs) in the partial, late‐acting reproductive isolation observed between the successional varieties. Fruit set was strongly reduced for backcross‐*incana* trees (35%–44% lower than fruit set of maternal trees of the two parental varieties), but more weakly reduced for the F_1_ trees (22%–33% lower), and pollen stainability was significantly reduced for pooled hybrid trees. This pattern of reduced hybrid fertility is similar to (but weaker than) that observed by Fishman and Willis (2001) in their test of chromosomal rearrangements versus DMIs as the cause of postzygotic reproductive isolating barriers between species of *Mimulus*. In both *Metrosideros* and *Mimulus*, the lower female fertility of second‐generation hybrids compared to F_1_s is consistent with fertility loss due to recessive, diploid DMIs (Fishman & Willis, 2001). Additional evidence for DMIs in *incana‐glaberrima* hybrids was suggested by patterns of seedling growth. Eight‐month‐old selfed seedlings of F_1_ trees were significantly stunted (43%–48% reduction in height) compared to those of the other tree classes, even with the negative effect of selfing on seedling height in all tree classes. This result was surprising given the lower inbreeding depression expected in hybrids. Selfing of F_1_ trees to produce F_2_ offspring represents an early opportunity for epistasis in mixed genomes to affect seedling traits (Falconer & Mackay, 1996). Lastly, beyond the “variety effect” on 2‐year‐old seedling heights, there were contrasting patterns between seedlings from the two hybrid classes (Figure 3). Although these interactions were not significant with the sample sizes available, they suggest the influence of gene interactions on the early growth of hybrid seedlings. DMIs are the major genetic source of inviability and sterility in hybrids (Coyne & Orr, 2004) and have been implicated as the cause of postzygotic isolation in a range of plant groups (e.g., Bomblies et al., 2007; Burke, Voss, & Arnold, 1998; Fishman & Willis, 2001; Jiang et al., 2000; Kubo, Yoshimura, & Kurata, 2011; Rieseberg, Sinervo, Linder, Ungerer, & Arias, 1996; Scopece, Widmer, & Cozzolino, 2008). Our results suggest that DMIs may play an important role in the early stages of ecological speciation in trees.

DMIs are thought to arise in hybrids from deleterious interactions between alleles at different loci that evolve independently and innocuously in the parental populations (Coyne & Orr, 2004), whether in allopatry (Orr, 1995) or parapatry (Gavrilets, 2004; Gavrilets, Li, & Vose, 2000; Kondrashov, 2003). In the case of *M. polymorpha*, divergent selection on vegetative, physiological, and life history traits on contiguous new and old lava flows may have resulted in the accumulation of incompatibilities affecting *incana‐glaberrima* hybrids. The two parental varieties are distinguished by vegetative traits with a predominantly additive genetic basis. Other than showing decreased fertility, hybrid trees were intermediate between *incana* and *glaberrima* for all traits found to differ between varieties in this study: fruit maturation time, speed of seed germination, and height of seedling offspring (i.e., early allocation to roots vs. shoots). Further, although phenology was not quantified, during the flowering period (predominantly April through mid‐July in both years), flowering overlapped strongly among the four tree types, with a trend of *glaberrima* flowering earlier during that period, *incana* later, and hybrids in between (E. Stacy and B. Paritosh, pers. obs.). Additionally, hybrids were intermediate on average at both the adult stage (100% of nine morphological traits that differed between the varieties) and seedling stage (eight of nine morphological traits measured in F_1_ seedlings produced through the crosses reported here; Stacy et al. (2016)). The intermediate nature of the hybrids in morphological and life history traits indicates that the quantitative traits that distinguish *incana* and *glaberrima* are heritable with largely an additive genetic basis (Stacy et al., 2016). The substantial number of differences between these varieties suggests divergence at a large number of loci, yet how these differences might interact to cause incompatibilities in hybrids, especially those affecting fertility, is unknown. Hybrid incompatibilities may result from negative interactions directly between alleles involved in adaptation to early and late‐successional environments (Bierne et al. 2011), or from the evolution of modifier alleles that ameliorate negative pleiotropic consequences of locally adapted alleles (Agrawal et al., 2011; Bierne et al. 2011). Pleiotropy of genes involved in local adaptation may be suggested by the strong genetic correlations observed among some leaf traits in these varieties (e.g., r ~ 1.0 ± 0.004 (SE) between leaf length and leaf width for *incana*, and 0.746 ± 0.005 between leaf length and petiole length for *glaberrima*; Stacy et al., 2016). Hybrid incompatibilities affecting fertility may have arisen also through endogenous selection on reproductive traits within the large, contiguous populations of these varieties, followed by partial coupling of these incompatibilities with those arising from differential adaptation of the parent taxa to their home environments (Smadja & Butlin, 2011; Bierne et al. 2011; Abbott et al., 2013).

### 4.3 Incomplete speciation between *incana* and *glaberrima*

In the case of the successional varieties of Hawaii's dominant tree, *M. polymorpha*, the dynamic nature of selection on active volcanoes may explain their isolation by only relatively weak barriers despite the presence of stronger barriers elsewhere within the Hawaiian *Metrosideros* radiation. The older island of O'ahu hosts multiple *Metrosideros* taxa (including species) that show significantly greater morphological and neutral genetic differentiation relative to that observed for *incana* and *glaberrima* on Hawai'i Island (E. Stacy and T. Sakishima, unpub. data). The greater neutral genetic isolation of these forms despite their sympatry with each other and the multiple varieties of *M. polymorpha* on O'ahu indicates that significant isolating barriers have arisen within this ~4‐myo radiation (Percy et al., 2008) under certain conditions. In contrast, the partial reduction in female and male fertility of *incana‐glaberrima* hybrids on Hawai'i Island indicates that these taxa are at an early stage of speciation. Weak reproductive isolation due to divergent selection, sometimes accompanied by weak neutral genetic differentiation, may be common and may not always proceed to speciation (Nosil, Harmon, & Seehausen, 2009). Instead, partial reproductive isolation may reflect a stable equilibrium between divergent selection and gene flow (McAllister, Sheeley, Mena, Evans, & Schlötterer, 2008; Nosil et al., 2009). *Incana* and *glaberrima* may fit the “stuck partway” view of speciation (Berlocher & Feder, 2002), with partial reproductive isolation stabilized by recurring cycles of differential purifying selection on new (and old) lava flows with each new eruption, followed by introgressive hybridization on intermediate‐aged flows (Stacy et al., 2016). The natural, recurring disturbance that generates this spatial and temporal variation in disruptive selection on these trees has a history longer than that of *Metrosideros* in the Hawaiian Islands; yet, today active volcanoes are found only on young Hawai'i Island where *incana* and *glaberrima* are especially abundant. Further work is needed to determine whether the loss of disruptive selection on the extinct volcanoes of the older Hawaiian Islands allows the accumulation of additional barriers between *incana* and *glaberrima* or the dissolution of the hybrid incompatibilities that partially isolate them on Hawai'i Island.

### 4.4 Merging studies above and below the species boundary

This study is unusual among studies of reproductive isolating barriers between plant lineages in finding only one significant isolating barrier between the taxa examined. The difference between the current study and most others is that the latter are predominantly conducted above the species boundary, where numerous isolating barriers are typically found. While such studies aid our understanding of how species boundaries are maintained, only in combination with examination at the early stages of population divergence can we uncover early arising barriers during speciation (Nosil, Vines, & Funk, 2005; Via, 2009). Early botanists recognized that ecologically differentiated plant populations represent intermediate stages in the speciation process (e.g., Clausen, 1951), and there is a sizeable body of the literature that examines the strengths and stages of reproductive isolating barriers within plant species, often in association with adaptation to contrasting environments. These studies of postzygotic reproductive isolation below the species boundary—or outbreeding depression (Price & Waser, 1979)—reveal partial isolation within species across environmental gradients or ecotones (Fenster & Galloway, 2000; Grindeland, 2008; Montalvo & Ellstrand, 2001; Waser, Price, & Shaw, 2000), even in trees (Goto, Iijima, Ogawa, & Ohya, 2011; Stacy, 2001). In such cases, outbreeding depression appears to result from the disruption of local adaptation; however, partial reproductive isolation between conspecific populations can result also from physiological or intrinsic causes such as underdominance (heterozygote disadvantage), chromosomal rearrangements, or the breakup of coadapted gene complexes (Edmands, 2002; Price & Waser, 1979). The outbreeding depression observed within plant species over moderate spatial scales is sometimes even accompanied by differential fertilization by pollen donors, which may reflect reinforcing selection against hybridization between genetically diverged parents (Waser, 1993 and references therein). In contrast with findings above the species level where individual isolating barriers are often strong and total isolation may be complete, patterns of reproductive isolation below the species boundary are often messy and dependent on the spatial scales of habitat heterogeneity and species dispersal (Edmands, 2002; Waser et al., 2000). Nonetheless, given that examinations of outbreeding depression within species reveal insights into the earliest stages of speciation (Edmands, 2002; Stacy, 2001; Waser et al., 2000), more studies below the species boundary are called for—as well as integration of the current literature on outbreeding depression (including recent conservation genetics literature (Scopece et al., 2010)—if reproductive barriers that arise early during speciation are to be understood.

## 5 Conclusions

We examined postpollination reproductive isolating barriers at an early stage of speciation between two ecologically diverged but hybridizing varieties of a dominant, long‐lived and highly dispersible tree species and found significant, partial postzygotic isolation in the form of reduced fertility of hybrids, especially backcross trees. *Incana* and *glaberrima* are morphologically distinct, co‐occurring forms of the hypervariable Hawaiian tree species, *M. polymorpha*, that appear to represent an early stage of ecological speciation driven by adaptation to new and old lava flows. Persistent environmental instability, coupled with tree‐specific traits that are expected to slow barrier formation, may hamper the evolution of further isolating barriers between these varieties. Examination of isolating barriers between these forms on older Hawaiian Islands is needed to determine whether the loss of disruptive selection by active volcanoes leads to the accumulation of further isolating barriers, the loss of hybrid incompatibilities, or no change in the strength of reproductive isolation between these incipient species.

## Conflict of interest

The authors have no conflict of interest.

## Data accessibility

Data available from the Dryad Digital Repository: http://dx.doi.org/10.5061/dryad.q0s20
