# Supplementary material for: Incipient ecological speciation between successional varieties of a dominant tree involves intrinsic postzygotic isolating barriers
Source: Ecol Evol. 2017 Mar 14;7(8):2501–12. doi: 10.1002/ece3.2867 (PMC5395442; doi:10.1002/ece3.2867)
Supplement: Supplementary file 1 [file ECE3-7-2501-s001.pdf]

APPENDIX:

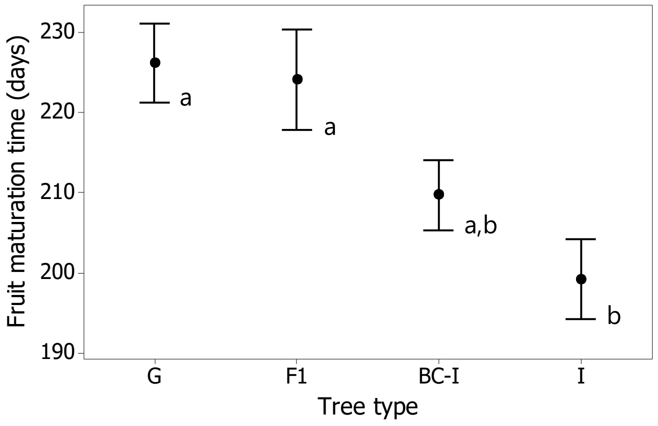

Figure S1. The duration of the fruit maturation period of two varieties and their hybrids at the study site; data are for hand- and open-pollinated flowers pooled. Mean (± 1 SE) number of days between hand-pollination and first mature fruit collection for 30 maternal trees of *glaberrima* (G), 15 F_1 trees (F1), 13 backcross-*incana* trees (BC-I), and 11 trees of *incana* (I). Groups with shared superscripts are not significantly different at $\alpha = .05$.

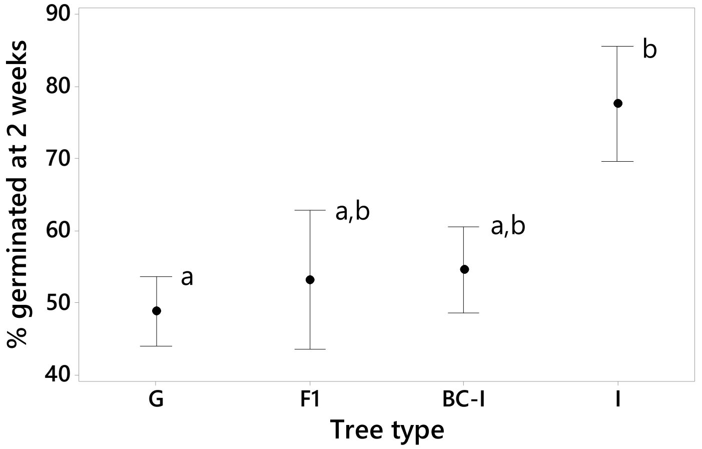

Figure S2. The mean proportion (± 1 SE) of total germinants that had germinated by two weeks post sowing of seeds from four maternal tree types: *glaberrima* (G; n = 30), F_1 hybrids (F1; n = 15), backcross-*incana* hybrids (BC-I; n = 13), and *incana* (I; n = 11). Seeds are from experimental outcrosses only, and sharing of superscripts indicates no significant difference at $\alpha = .05$.

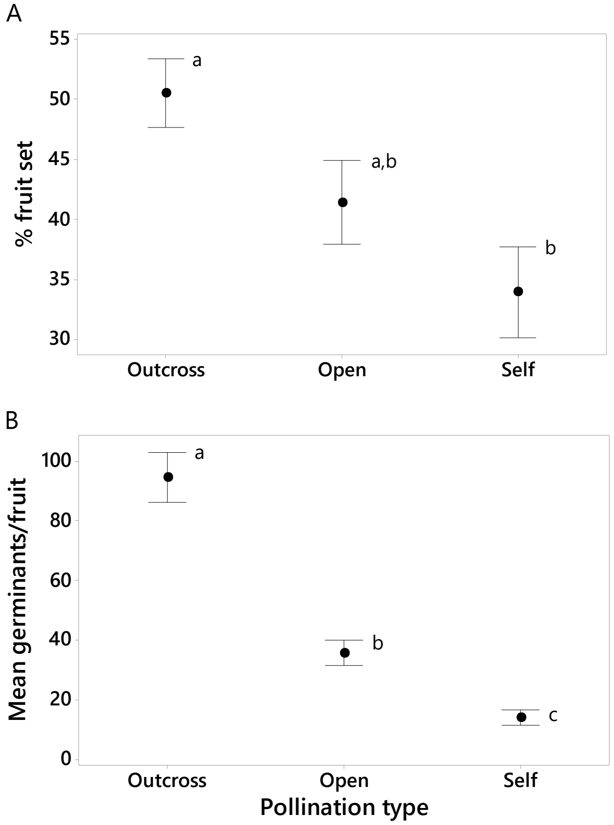

Figure S3. Comparison of three pollination treatments. A) Mean (± 1 SE) percentage fruit set for outcross-, open-, and self-pollinated flowers for 62 hybrid-zone trees for which all three pollination treatments were done, and B) Mean (± 1 SE) number of germinants per fruit for outcross-, open-, and self-pollinated flowers for 43 hybrid-zone trees for which fruits from all three pollination treatments were available. Groups with shared superscripts are not significantly different at α = .05.

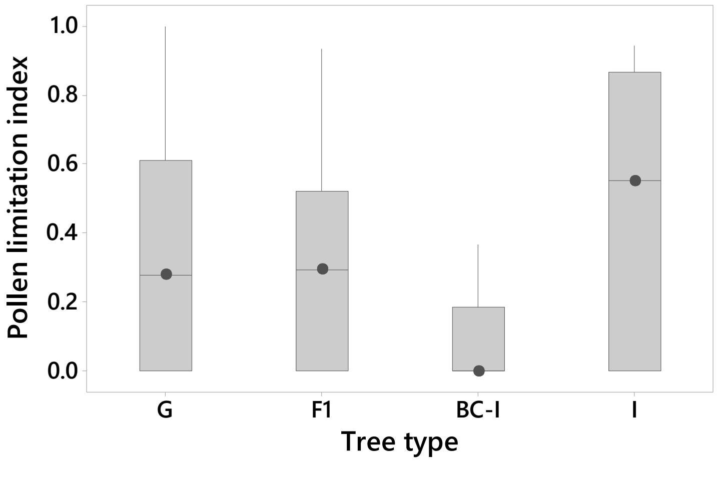

Figure S4. Median, quartiles and range of the pollen limitation index (PLI) for each of four tree types: *glaberrima* (G; n = 28), F_1 hybrids (F1; n = 13), backcross-*incana* hybrids (BC-I; n = 13), and *incana* (I; n = 11). The trend of a lower PLI for backcross-*incana* trees was not statisti stically significant (P = .112).
